# Loss of heterozygosity on chromosome 16q increases relapse risk in Wilms’ tumor: a meta-analysis

**DOI:** 10.18632/oncotarget.20191

**Published:** 2017-08-11

**Authors:** Zhenyu Pan, Hairong He, Lina Tang, Qingting Bu, Hua Cheng, Anmin Wang, Jun Lyu, Haisheng You

**Affiliations:** ^1^ Clinical Research Center, The First Affiliated Hospital of Xi'an Jiaotong University, Xi’an, Shaanxi, 710061, China; ^2^ Department of Pharmacy, Xi’an Jiaotong University Affiliated Children’s Hospital, Xi’an, Shaanxi, 710003, China; ^3^ Department of Pharmacy, The First Affiliated Hospital of Xi'an Jiaotong University, Xi’an, Shaanxi, 710061, China; ^4^ Department of Genetics, Northwest Women’s and Children’s Hospital, Xi’an, Shaanxi, 710061, China

**Keywords:** Wilms’ tumor, LOH 16q, relapse, meta-analysis

## Abstract

Wilms’ tumor (WT) is the most frequent malignant renal tumor in children. The survival rate is lower in patients with recurrence, and the factors that influence relapse in WT are not fully understood. Loss of heterozygosity on chromosome 16q (LOH 16q) has been reported to be associated with the relapse in WT, but this remains controversial. We performed a meta-analysis to clarify this. PUBMED, EMBASE, and the Cochrane Library were searched up to March 17, 2017. Ten studies involving 3385 patients were ultimately included in the meta-analysis. The meta-analysis showed that LOH 16q was significantly associated with the relapse in WT (relative risk [RR] = 1.74, 95% confidence interval [CI] = 1.43–2.13, *P* < 0.00001; hazard ratio [HR] = 1.76, 95% CI = 1.38–2.24, *P* < 0.00001). No significant heterogeneity among studies or publication bias was found. Sensitivity analysis showed omitting one study in each turn could not change the results. Subgroup analysis based on two studies indicated LOH 16q was more effective on elevated replase risk in patients with favorable-histology WT (RR = 2.52, 95% CI = 1.68–3.78, *P* < 0.00001; HR = 2.99, 95% CI = 1.84–4.88, *P* < 0.0001) but further work are needed to confirm this. These findings confirm that LOH 16q increased the relapse risk in WT, but more studies are required to further assess the association between LOH 16q and WT relapse among different subgroups.

## INTRODUCTION

Wilms’ tumor (WT) is the most common malignant renal tumor in children [1]. Treatments of WT have improved significantly over the past 40 years, and approximately 90% of patients now achieve a long survival. This progress has been attributed to combining the clinical stage and histological type [2]. However, WT remains to be conquered completely. Only approximately 50% of patients who relapse will survive [3, 4], and hence it is important to identify the prognostic factors associated with the relapse in patients with WT, and more intense treatment may need to be added early to patients with worse prognostic factors.

Some studies have found loss of heterozygosity on chromosome 16q (LOH 16q) to be involved in malignant progression of various tumor types, including WT and those of the breast, prostate, and liver [5–8]. LOH 16q is present in 20–30% of WT patients. The National Wilms Tumor Study (NWTS) first proposed the hypothesis that WT with LOH 16q is associated with relapse based on a study of 232 cases of WT [9], and the findings of several other studies support this conclusion [10, 11]. However, other studies have failed to find a significant role of LOH 16q in recurrence among WT patients [12–15], and so it is still unclear whether LOH 16q is associated with WT relapse.

Our goal was to determine the association between LOH 16q and relapse in WT by conducting a meta-analysis. This is the first meta-analysis to determine this relation, and the findings might further help to predict the prognosis and improve the treatment of WT.

## RESULTS

### Study selection

**Figure 1:**
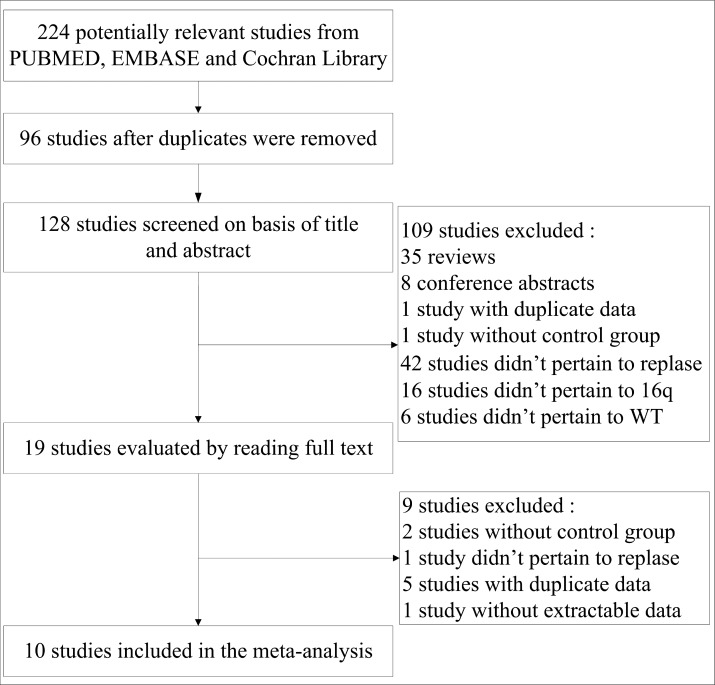
Flow diagram for study selection

In total, 224 relevant studies were identified using PUBMED, EMBASE, and the Cochrane Library, of which 96 duplicates were removed. Scanning the titles and Abstracts resulted in a further 109 studies being excluded because they did not meet the inclusion criteria. The remaining 19 studies were further evaluated by reading the full texts, which resulted in 9 studies being excluded due to a lack of useful data or the presence of duplicate data. Therefore, 10 studies [9–11, 13–19] involving 3385 patients were ultimately included in the meta-analysis. Figure 1 shows the process of study selection.

### Study characteristics and quality assessment

The 10 included studies were reported on between 1994 and 2017, and all of them were cohort studies. Four of them involved international collaborations [14, 15, 17, 19], five were performed in Europe [9–11, 13, 18], and one was performed in North America [16]. The characteristics of the included studies are presented in Table 1, including the number of patients and their ages, the follow-up period, method to detect LOH 16q, and the quality scores. The quality scores were determined with reference to the Newcastle-Ottawa Scale (NOS) about cohort studies: three studies [14, 15, 19] were of high quality while the other seven [9–11, 13, 16–18] were of moderate quality. The mean with standard deviation of NOS score is 5.8 ± 0.92.

**Table 1:** Characteristics of included studies

Study	Country	Patients	Age	Follow-up	Method to Detect LOH 16q	NOS score
Grundy PE 1994	USA and Canada	206 patients with favorable-histology WT	Not known	Median follow-up durations in LOH and non-LOH groups of 1.3 and 1.4 years, respectively	PCR	6
Klamt B 1998	Germany, Austria, and Switzerland	73 patients with WT	Mean 3.48 years	Not known	PCR	5
Grundy RG 1998	UK	40 patients with sporadic WT	Not known	At least 7 years	PCR	5
Skotnicka KG 2000	Poland	66 patients with WT	Median 39 months, range 2 days to 13 years	Median 42 months, range 14 to 139 months	PCR	5
Kullendorff CM 2003	Sweden	39 patients with WT	Mean 4.2 years, range 5 months to 15 years	Range 7 to 160 months	Not known	5
Grundy PE 2005	USA, Canada, Australia, New Zealand, Switzerland, and the Netherlands	2021 patients younger than 16 years at diagnosis with specific WT	Younger than 16 years	4 years	PCR	7
Messahel B 2009	UK	426 patients with favorable-histology WT	Not known	4 years	Microsatellite markers	6
Spreafico F 2012	Italy	125 patients with nonanaplastic unilateral WT of stages I to IV	Median 40 months, range 1 to 172 months	Mean 73 months, range 35 to 97 months	Microsatellite markers	5
Chagtai T 2016	26 countries: 24 in Europe, 1 in Australia, 1 in South America	586 patients with WT of stages I to IV	Range 6 months to 18 years	Median 68 months	Multiplex Ligation-Dependent Probe Amplification	7
Fernandez CV 2017	the United States, Canada, Australia, New Zealand, and Israel	116 patients with very low risk WT (defined as stage I favorable histology WT with nephrectomy weight < 550 g and age at diagnosis < 2 years)	11.5 months: 0.1 to 23 months	80 months: 5 to 97 months	Multiplex Ligation-Dependent Probe Amplification	7

Abbreviations: LOH, Loss of heterozygosity; WT, Wilms’ tumor.

### LOH 16q and relapse

**Figure 2:**
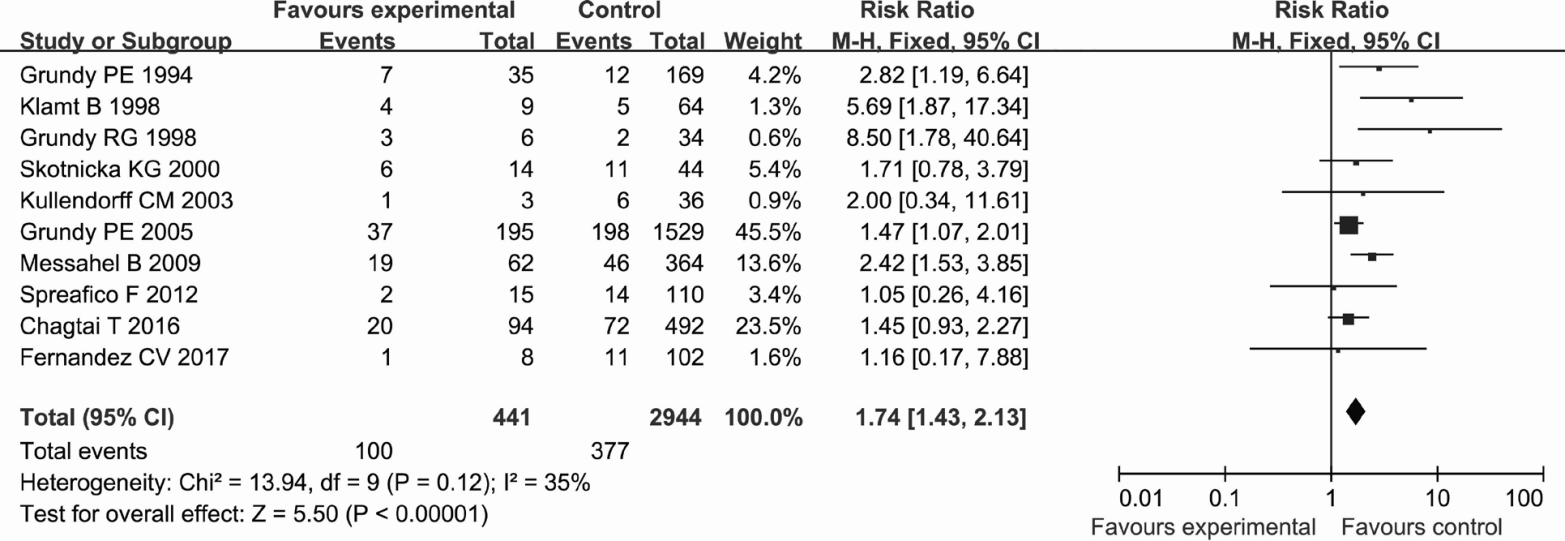
Forest plot of the association between LOH 16q and the relapse of WT using the RR as the effect measure

The estimated relative risk (RR) for the association between exposure to LOH 16q and WT relapse is presented in Figure 2. All of the included studies mentioned the RR for relapse. A fixed-effects model was used to pool the data. The pooled estimate showed that the LOH 16q was significantly associated with a higher risk of the relapse compared with that in the control group (RR = 1.74, 95% confidence interval [CI] = 1.43–2.13, *P* < 0.00001). There was no significant heterogeneity among the studies for this outcome (*P*_heterogeneity_ = 0.12, *I*^2^ = 35%).

**Figure 3:**
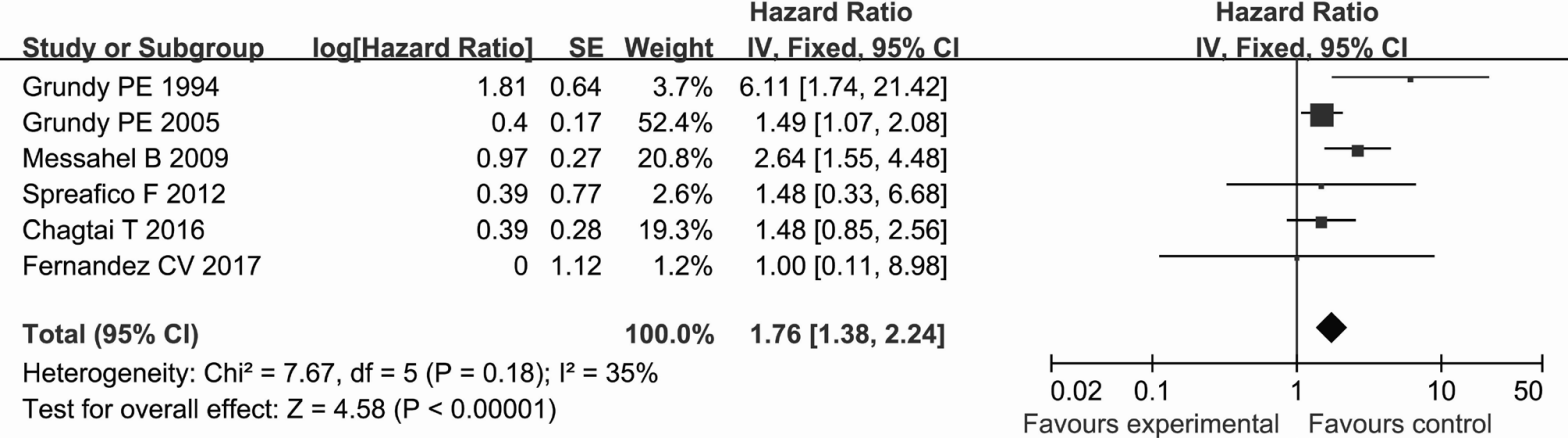
Forest plot of the association between LOH 16q and the relapse of WT using the HR as the effect measure

The estimated hazard ratio (HR) for the association between exposure to LOH 16q and WT relapse is shown in Figure 3. Six studies investigated the relapse risk of WT based on analyzing the HR. The fixed-effects model was used to calculate the pooled HR due to the absence of significant heterogeneity among the studies (*P*_heterogeneity_ = 0.18, *I*^2^ = 15%). The pooled estimate showed LOH 16q to be significantly associated with a higher risk of WT relapse (HR = 1.76, 95% CI = 1.38–2.24, *P* < 0.00001).

**Figure 4:**
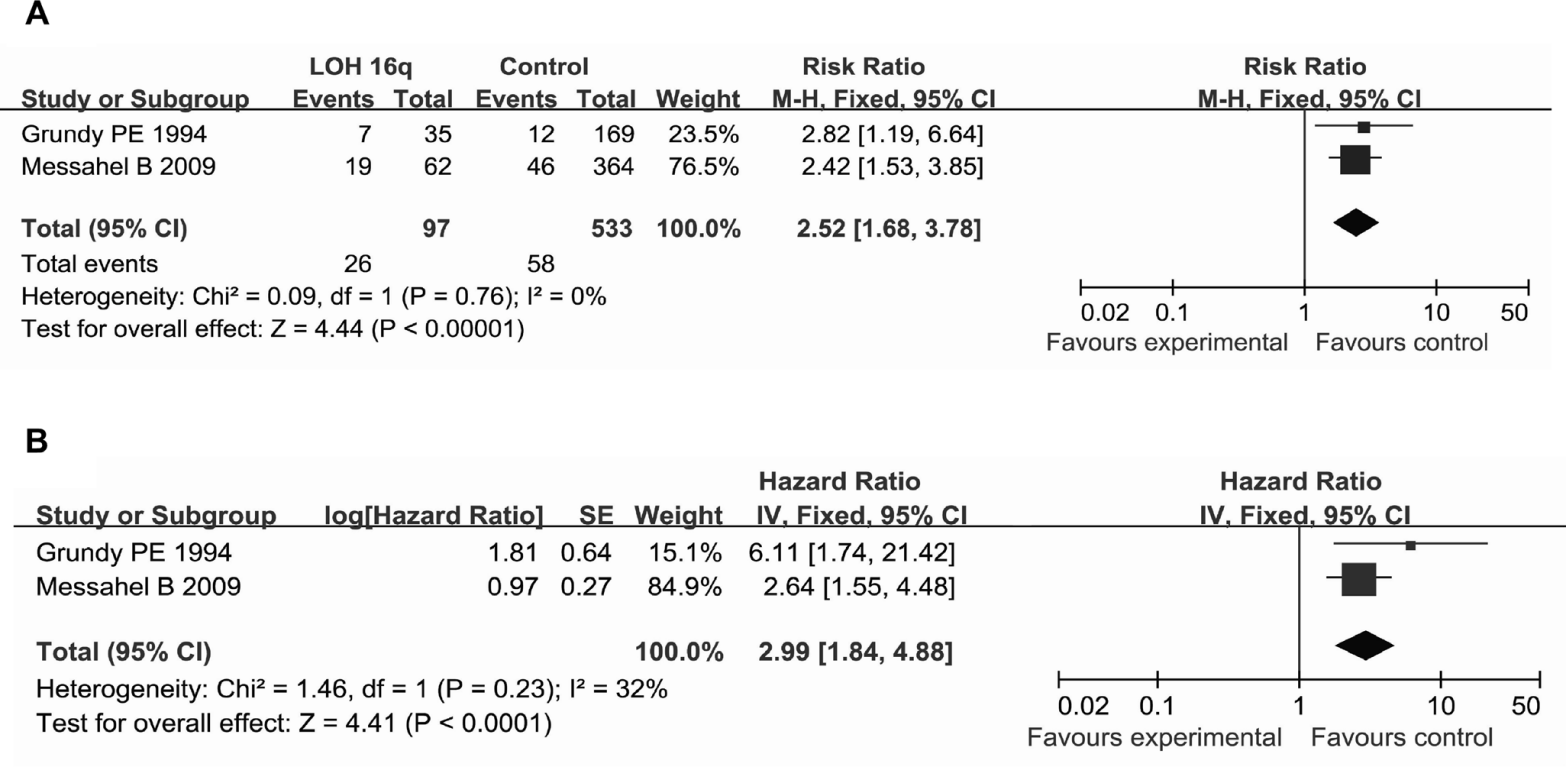
Forest plot of the association between LOH 16q and the relapse in patients with favorable-histology WT (**A**) using the RR as the effect measure; (**B**) using the HR as the effect measure.

Two studies enrolled patients with favorable-histology WT. Subgroup analysis was performed by patients with favorable-histology WT. The pooled RR for favorable-histology WT was 2.52 (95% CI = 1.68–3.78, *P* < 0.00001, *P*_heterogeneity_ = 0.76, *I*^2^ = 0%) (Figure 4A). The pooled HR for favorable-histology WT was 2.99 (95% CI = 1.84–4.88, *P* < 0.0001, *P*_heterogeneity_ = 0.23, *I*^2^ = 32%) (Figure 4B). The results indicated that LOH 16q was more effective on elevated replase risk in patients with favorable-histology WT.

### Publication bias

**Figure 5:**
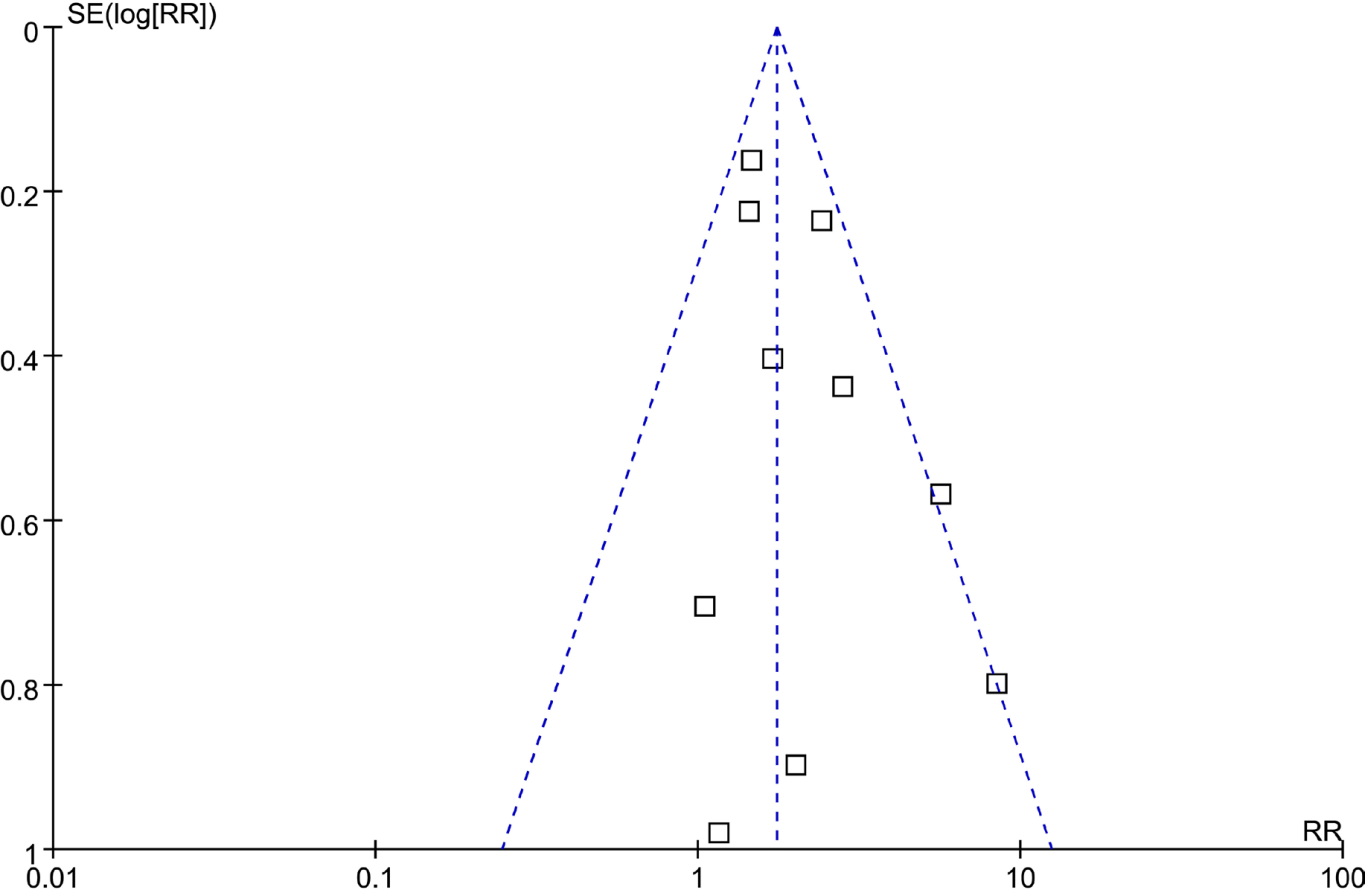
Funnel plot for detecting publication bias

Funnel plot, Begg’s test and Egger’s test were used to investigate publication bias in the included studies. Visual inspection of the funnel plot did not identify substantial asymmetry (Figure 5), indicating no evidence of substantial publication bias. The Begg’s and Egger’s tests also confirmed the absence of significant publication bias among the included studies (Begg’s test: *P* = 0.283; Egger’s test: *P* = 0.053).

### Sensitivity analysis

Sensitivity analysis showed that a significant association between LOH 16q and relapse in WT was still observable even when any single study was removed (Table 2 and Table 3), which mean the stability of the result that LOH 16q was associated with increased replase risk.

**Table 2:** Sensitivity analysis of association between LOH 16q and WT relapse risk for the RR by omitting one study in each turn

Study omitted	Pooled RR	95% CI	*P*
Grundy PE 1994	1.7	1.38–2.08	< 0.00001
Klamt B 1998	1.7	1.39–2.08	< 0.00001
Grundy RG 1998	1.69	1.38–2.07	< 0.00001
Skotnicka KG 2000	1.75	1.42–2.14	< 0.00001
Kullendorff CM 2003	1.74	1.43–2.13	< 0.00001
Grundy PE 2005	1.98	1.53–2.55	< 0.00001
Messahel B 2009	1.64	1.31–2.04	< 0.0001
Spreafico F 2012	1.77	1.45–2.16	< 0.00001
Chagtai T 2016	1.83	1.47–2.29	< 0.00001
Fernandez CV 2017	1.75	1.44–2.14	< 0.00001

**Table 3:** Sensitivity analysis of association between LOH 16q and WT relapse risk for the HR by omitting one study in each turn

Study omitted	Pooled HR	95% CI	*P*
Grundy PE 1994	1.67	1.31–2.14	< 0.0001
Grundy PE 2005	2.1	1.48–2.99	< 0.0001
Messahel B 2009	1.58	1.2–2.07	0.001
Spreafico F 2012	1.77	1.38–2.25	< 0.00001
Chagtai T 2016	1.83	1.4–2.4	< 0.0001
Fernandez CV 2017	1.77	1.39–2.26	< 0.00001

## DISCUSSION

LOH 16q is present in 20–30% of WT cases [9], and a recent study found that the mean frequency of LOH 16q was 15.1% (95% CI = 12.9–17.2%) [20]. A LOH 16q status has been associated with WT relapse and might consequently play an important role in determining the optimal treatment [5, 21]. This information is being used to stratify patients within NWTS therapeutic protocols to warrant more intensive early drug regimens [19, 22]. However, other studies have found no significant association between LOH 16q and WT relapse [12–15], and hence this relationship remains controversial. The present meta-analysis is, to the best of our knowledge, the first to evaluate whether LOH 16q is associated with the relapse in WT.

Ten cohort studies were included in this meta-analysis. Although cohort studies have many methodological shortcomings in comparison with randomized controlled trials (RCTs), we included cohort studies in the inclusion criteria rather than RCTs based on feasibility. RCT is a kind of intervention trial, and intervention measures involving LOH in patients are technically difficult to implement and they have major ethical problems. Furthermore, it was found that there were no RCTs investigating this topic in the initially searched 224 studies from PUBMED, EMBASE, and the Cochrane Library.

Our meta-analysis revealed that LOH 16q was significantly associated with the relapse in WT using the RR as the effect measure, and that there was no significant heterogeneity among the included studies. Nevertheless, relapses are time-to-event outcomes, and the RR only measures the number of relapses without accounting for when they occur [23]. This may introduce bias into the results and lead to inappropriate conclusions when analyzing time-to-event outcomes such as relapses. In contrast, the HR summarizes the difference between two Kaplan-Meier curves, and represents the overall reduction in the risk of time-to-event outcomes over the follow-up period among patients [24]. It is still defective only to use the HR as the effect measure due to the lnHRs and their standard errors of some included studies were estimated according to the methods of Tierney et al., which also may introduce bias into the results and inappropriate conclusions. We therefore used the RR and the HR as the effect measures to analyze relapses in WT in order to check whether the conclusions were sensitive to different effect measures. It was found that there was still a significant association between LOH 16q and WT relapse when using the HR, and that there was no significant heterogeneity among the included studies. Interestingly, the sample size of 10 included studies involving 3385 cases was imbalanced, since the study of Grundy et al. [19] involved 1724 cases and the weight of this study reached 45.5% and 52.4% (Figures 2 and 3), which imply this study of Grundy et al. may have a greater impact on the results of this meta-analysis. However, the conclusions did not change when we conducted a sensitivity analysis that involved the exclusion of individual studies, even when the study excluded was that of Grundy et al. [19]. In addition, no publication bias was detected by funnel plot and Begg’s and Egger’s tests in our study. These results, no significant heterogeneity among studies and publication bias and the results of sensitivity analysis, mean the meta-analysis are stable and credible.

The mechanism by which LOH 16q increases the recurrence risk in WT may involve the effects of LOH 16q on certain tumor-associated genes such as E2F4, COX4 [25], and CTCF [26, 27]. It is well known that gene distributions vary with race. The 10 studies included in the present analysis were mainly from Europe and North America, and although there are racial variations in these areas, the dominant race is Caucasian. A study that included 206 cases from North America [16] found the frequencies of LOH 16q to be 10%, 19%, 24%, 13%, and 14% in WT of stages I, II, III, IV, and V, respectively. In contrast, the frequencies of LOH 16q were 8%, 20%, 57%, and 0% in WT of stages I, II, III, and IV, respectively, in a study including 30 cases from China [28]. We supposed that factors such as race and tumor stage may affect the distribution of LOH 16q and further affect the relapse risk of WT. Unfortunately, the data available from the included studies were insufficient to perform a subgroup analyses based on race or tumor stage. Moreover, information regarding subclasses including bilateral⁄unilateral and age could not be extracted for most of the included studies to carry out more detailed subgroup analysis. These factors represent limitations of the present meta-analysis, and so future studies need to further explore the significance of LOH 16q in WT replase in various subgroups. Two studies mentioned favorable-histology WT and a subgroup analysis was performed based on the factor. Although subgroup analysis indicated that LOH 16q was more effective on increasing replase risk in patients with favorable-histology WT, further work are needed to confirm this duo to few studies and small sample sizes.

In summary, we have found that LOH 16q increased the relapse risk in WT. The high statistical power of this study has provided more precise and reliable estimates than those reported previously. This information will be helpful when applying early preventive measures in clinical settings according to the existence of LOH 16q. However, more studies are recommended for further assessing the role of LOH 16q in increasing the relapse risk among different subgroups of WT and for identifying the underlying mechanisms.

## MATERIALS AND METHODS

### Search strategy and selection criteria

We searched PUBMED, EMBASE, and the Cochrane Library in accordance with the PRISMA statement up to March 17, 2017 [29] using the following search terms: **(**Wilms’ tumor OR renal embryoma OR nephroblastoma) AND (16q OR loss of heterozygosity in 16q OR LOH of 16q)**.** The studies were selected based on the following criteria: (1) cohort studies involving patients with WT, (2) the exposure of interest was LOH 16q, (3) the outcome of interest was relapse, and (4) reporting total numbers and replase numbers of LOH 16q cases and controls (non-LOH 16q) or the natural logarithm of HR (lnHR) and its standard error, or other data sufficient to calculate them. Studies with overlapping data, reviews, nonclinical studies, case observations, and letters were excluded from the present analysis.

### Data extraction

Two reviewers independently screened the titles and Abstracts of all studies identified by applying the search strategy and assessed these studies using predetermined selection criteria. The full texts of all potentially relevant studies were retrieved for detailed review, and disagreements were resolved by consensus with the third reviewer. Two reviewers used a predefined data collection form to independently extract the following data from each included study: name of first author, year of publication, country, characteristics of patients, age, duration of follow-up, method to detect LOH 16q, total numbers and replase numbers of LOH 16q cases and controls, and lnHR and its standard error. If the lnHR and its standard error were not reported, they were estimated according to the methods of Tierney et al. [23].

### Quality assessment

The quality assessment of each study was assessed based on the NOS about cohort studies [30]. Two reviewers independently performed a methodological quality assessment, and disagreements were resolved by the third reviewer. The included studies were evaluated based on the aspects of selection (4 points), comparability (2 points), and outcomes (3 points), with total scores of 0–3, 4–6, and 7–9 points indicating low, moderate, and high quality, respectively. Average score was presented as mean ± standard deviation.

### Statistical analysis

Statistical analysis was performed using Cochrane RevMan 5.1 software. Categorical variables were compared using the RR and HR, and 95% CI values were calculated. The *I*^2^ test and Cochran’s *Q*-test were applied to estimate the heterogeneity among the studies. The heterogeneity was considered to be significant if the *P* value of the *Q*-test was < 0.05 or *I*^2^ was ≥ 0%. Data with significant heterogeneity were studied using a random-effects model, while a fixed-effects model was applied to other data. Funnel plot and Begg’s test [31] and Egger’s test [32] were used to assess publication bias. A *P* < 0.05 was considered to be indicative of statistical significance. A sensitivity analysis was performed to test the stability of the results by omitting one study in each turn.
